# The effect of vitamin E and aspirin on the uterine artery blood flow in women with recurrent abortion: A single-blind randomized controlled trial

**Published:** 2017-10

**Authors:** Elaheh Mesdaghinia, Behnaz Mohammad-Ebrahimi, Fatemeh Foroozanfard, Hamid Reza Banafshe

**Affiliations:** 1 *Department of Obstetrics and Gynecology, Kashan University of Medical Sciences, Kashan, Iran.*; 2 *Physiology Research Center, Kashan University of Medical Sciences, Kashan, Iran.*; 3 *Department of Pharmacology, School of Medicine, Kashan University of Medical Sciences, Kashan, Iran.*

**Keywords:** Recurrent abortion, Aspirin, Vitamin E, Pulsatility index

## Abstract

**Background:**

Recurrent spontaneous abortion has high incidence rate. The etiology is unknown in 30-40%. However high uterine artery resistance is accounted as one of the recurrent abortion reasons.

**Objective:**

The objective of the current study was to determine the impacts of vitamin E and aspirin on the uterine artery blood flow in women having recurrent abortions due to impaired uterine blood flow.

**Materials and Methods:**

This randomized clinical trial was conducted on 99 women having uterine pulsatility index (PI) more than 2.5 and the history of more than two times abortions. The candidates were categorized into three groups; receiving aspirin, only vitamin E, and aspirin+vitamin E. After 2 months, uterine PIs were compared with each other.

**Results:**

All drug regimens caused an enhancement in uterine perfusion with a significant decline in uterine artery PI value. The women receiving vitamin E in accompanied with aspirin had the least mean PI of the uterine artery (p<0.001). The total average PI score of the right and left uterine arteries in groups receiving vitamin E in accompanied with aspirin was lower than the two counterparts significantly (p<0.001).

**Conclusion:**

Vitamin E, aspirin and especially their combination are effective in improving uterine artery blood flow in women with recurrent abortion due to impaired uterine blood flow. More well-designed studies are needed to find out whether the enhancement of uterine perfusion may lead to a better pregnancy outcome.

## Introduction

Recurrent spontaneous abortion is defined as at least two consecutive abortions that are accounted for 2-5%. Although the etiology is unknown in 30-40%, there has been a consensus that it has multifactorial reasons either maternal or fetal (1). Some alterations occur in uterine perfusion during normal menstruation so that the uterine blood flow may be increased during luteal phase Impaired uterine blood flow disrupts embryo implementation. Irrespective of other etiological factors, impaired uterine perfusion is mainly accounted for spontaneous abortion (2).

The results have shown that uterine blood flow resistance is higher during the mid-luteal phase in women with recurrent abortions in comparison with normal women (3-5). Doppler ultrasound with an assessment of the pulsatility index (PI) as a value reflecting downstream resistance to blood flow has become widely accepted when monitoring high risk pregnancy. A relationship between increased impedance in uterine artery and poor pregnancy outcome has been reported by several studies (6-8). Some therapeutic approaches consisting of nitric oxide, sildenafil, and low dose aspirin have been propounded to improve uterine perfusion in women with recurrent spontaneous abortions (9). 

Aspirin is as the sort of non-steroid anti-inflammatory drugs whose target is cyclo-oxygenase enzyme that is a key element in arachidonic acid metabolism to prostanoids. Various studies have been carried out concerning aspirin in pregnancy. Recently, it has been demonstrated that the women who received low dose aspirin before the 16th wk of pregnancy had the experience of a significant decrease in prenatal mortality, intra-uterine growth retardation, preterm labor, and preeclampsia rates (10). 

A study conducted by Lazzarin and colleagues on 60 women with the history of recurrent spontaneous miscarriages concerning prescribing aspirin per se or plus omega 3 showed significant improved uterine blood flow leading to decreased spontaneous abortion rates due to impaired uterine perfusion (4). It has also been reported that prescribing aspirin in conjunction with heparin decreased significantly spontaneous abortion during the first trimester of pregnancy, but the study did not show how aspirin in conjunction with heparin worked to lead to this result. Moreover, the study demonstrated that prescribing aspirin solely did not produce the desired effect on reducing abortion (11).

Additionally, vitamin E deficiency is associated with increased peroxidase and aldehyde levels in various tissues (12). Recently it has been shown that vitamin E not only improved the function of the ovaries but also increased endometrial thickness; therefore, the chance of fertility would be increased whereas the rate of abortion due to decreased endometrial thickness would be diminished (13).

Giving that there have not been conducted studies on the effects of vitamin E and aspirin on the uterine blood flow and decreased abortion rate, the present study attempts to investigate the effects of vitamin E and aspirin on the uterine blood flow in women having the history of recurrent spontaneous abortion due to impaired uterine blood supply.

## Materials and methods

**Study population**

In this prospective single-blind randomized clinical trial, a total of 118 women with the history of recurrent spontaneous abortion owing the impairment of uterine blood supply with age between 21 and 41 who referred to Shabih-khani hospital, Kashan University of Medical Sciences, Iran, between May 2015 and March 2016 were enrolled in the study.

The clinical criteria for inclusion were the occurrence of two or more previous, first trimester abortion, not receiving hormonal methods of birth control during last three months, not using intrauterine devices, not receiving aspirin and vitamin E during the last week before the study. The exclusion criteria included not attending to fulfill follow-up ultrasound and being pregnant during the treatment. All patients underwent an accurate screening to exclude all the accepted recurrent abortion etiological factors such as thyroid function tests, antiphospholipid antibody levels, thrombophilia, thyroid anatomical abnormalities, and genetic tests were carried out. 

**Sample size**

**Figure 1:**
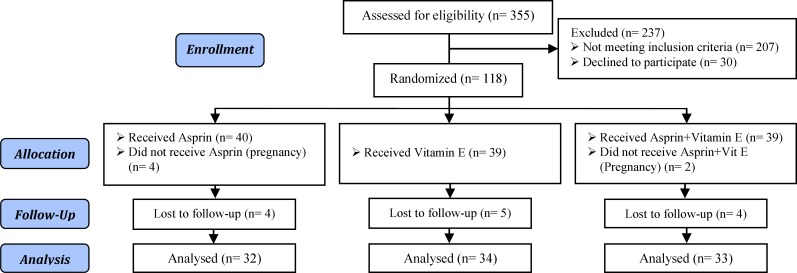
Consort flow chart of clinical trial

The sample size was calculated based on a similar study with considering type1 error 0.05 and second type error 20%, d= 0.35 (4). According to the study design and estimation of 20% loss to follow up, the sample size in each group of samples obtained 30. In the present study, 118 women with the history of more than two times abortions were investigated. 6 and 13 women have excluded from the study due to being pregnant and lack of having follow up; respectively. Hence, 99 were studied so that 32 received aspirin, 34 vitamin E, and 33 aspirin+ vitamin E (Figure 1).

**Measurements**

The studied women were undergone the examination and consultation by an obstetrician. After ruling out other reasons for spontaneous abortion, the women were referred to an ultrasound center. The ultrasound examination was performed using the color Doppler ultrasound machine (Acusan Antares Siemens, Germany equipped with a 6.5 MHz (5-7.5) transvaginal micro convex probe. The women underwent Doppler uterine ultrasonography in order to find those having uterine PI>2.5 and ultimately the selected women were categorized into three groups. The first group received aspirin 100 mg daily and placebo. The second one received vitamin E 400 mg daily and placebo. The third one was given aspirin and vitamin E daily. Block randomization method was used in the form of six and nine blocks. After initial registration and considering inclusion and exclusion criteria, every patient was allocated to the special group and the determined treatment protocol was prescribed for her.

The studied sample followed the prescription for two months. Doppler uterine ultrasonography was done in the luteal phase of the third month again without interrupting the treatment. It is important to mention that Doppler ultrasonography was performed by an ultrasonography specialist blinded to the study. The obtained data were recorded in a questionnaire. Before entering the study, the patients had detailed information about the study. 

**Ethical consideration**

The study received ethics approval from the ethical committee of Kashan University of Medical Sciences (IR.KAUMS.1394.28). Written informed consents were obtained from all participants.

**Statistical analysis**

Data were expressed as mean±SD. The analysis was done using Statistical Package for the Social Sciences, version 17.0, (SPSS Inc, Chicago, Illinois, USA). After obtaining data; firstly, the statistical measures in the three groups were calculated. Secondly; using statistical tests as paired t-test, Chi-square test, and ANOVA (Tukey´s, Post-hoc), the three groups were compared with each other (14, 15). Statistical significant was considered for p<0.05.

## Results

The results showed that the three groups in terms of background and confounding variables like age, number of pregnancies, deliveries, abortions and live births were not significantly different. The mean age was 30.88±5.5 yr. According to the ANOVA results, there were insignificant statistical differences among the groups concerning age ranges (p=0.987). The majority had 3 pregnancies and those having 2 pregnancies were on the next rank. Chi-square test showed that there were no significant differences statistically in terms of the number of pregnancies (p=0.327). Most of the women had 2 abortions. The number of abortions in the group receiving vitamin E and aspirin, the group receiving only vitamin E, and the group given only aspirin were 75.8%, 58.8%, and 53.1%; respectively. Chi square showed no comprehensible differences statistically regarding the number of abortions (p=0.25). 

**Figure 2:**
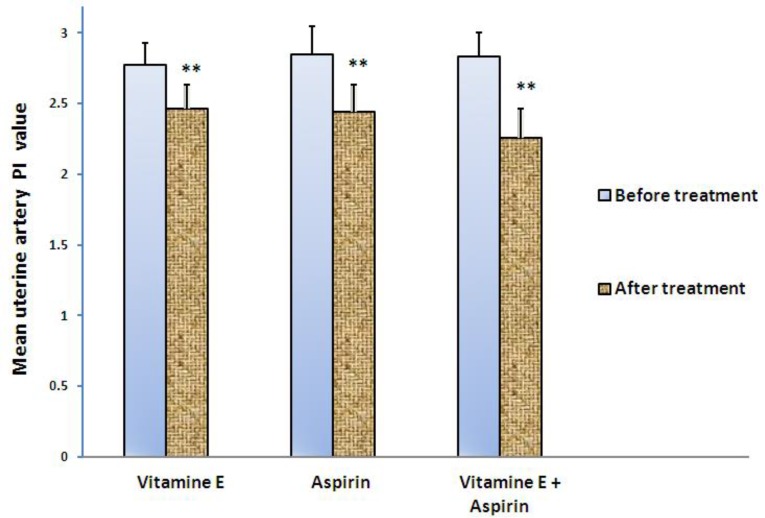
The mean uterine pulsatility index values before and after two months of therapy with aspirin (100mg/day), vitamin E (400mg/ day) and their combination. Data were expressed as mean ± SD. P-value of 0.05 was considered statistically significant.

As shown in table I, no significant differences were recorded between groups in terms of mean PI values before treatment. All treatments (aspirin (100 mg/day), vitamin E (400 mg/ day) and their combination) caused an enhancement of uterine artery blood flow as reflected in PI values that were significantly lower than PI values before treatment (Figure 2). The mean PI of the uterine artery in the group receiving vitamin E together with aspirin was decreased more than that of the other treatment groups (2.3 vs. 2.41 and 2.57. p<0.001). In other words, the alterations in the mentioned group were obvious. Then, the group receiving aspirin and finally the group receiving vitamin E had less differences regarding the mean PI; consecutively.

**Table I:** The difference of mean and standard deviation of uterine artery pulsatility index in three groups before and after the intervention

**Group**			**pulsatility index** *	**difference of mean**	**difference of S.D**	**t**	**df**	**p-value** **
Vitamin E+ Aspirin								
	Right uterine artery			0.4	0.21	10.68	32	<0.001
		Before	2.65 ± 0.33					
		After	2.25 ± 0.21					
	Left uterine artery			0.28	0.25	0.53	32	<0.001
		Before	2.65 ± 0.3					
		After	2.36 ± 0.22					
	Average two arteries			0.34	0.2	9.85	32	<0.001
		Before	2.65 ± 0.29					
		After	2.30 ± 0.2					
Vitamin E								
	Right uterine artery			0.04	0.06	0.03	33	<0.001
		Before	2.70 ± 0.34					
		After	2.60 ± 0.32					
	Left uterine artery			0.1	0.12	5.03	33	<0.001
		Before	2.74 ± 0.33					
		After	2.54 ± 0.31					
	Average two arteries			0.07	0.07	6.09	33	<0.001
		Before	2.72 ± 0.3					
		After	2.57 ± 0.28					
Aspirin								
Right uterine artery				0.32	0.15	11.66	31	<0.001
		Before	2.70 ± 0.33					
		After	2.38 ± 0.25					
	Left uterine artery			0.23	0.22	5.86	31	<0.001
		Before	2.67 ± 0.3					
		After	2.43 ± 0.31					
	Average two arteries			0.27	0.13	11.45	31	<0.001
		Before	2.68 ± 0.27					
		After	2.41 ± 0.24					

* Data presented as mean±SD.

** t-test

## Discussion

The findings of the present study show that uterine arteries PI values in women receiving vitamin E, aspirin, and vitamin E plus aspirin were decreased significantly. Moreover, the results demonstrated that the differences found in the mean PIs of the right, left, and both uterine arteries in the group receiving aspirin and vitamin E were more significant than the ones reported in the other groups. These therapeutic regimes have demonstrated to be important enhancer of uterine perfusion by improving uterine artery blood flow velocity. These findings will be clinically important since a growing body of evidences support the hypothesis that a sufficient uterine perfusion has a critical role in the success of pregnancy (4, 16 and 17).

A study carried out by Lazarrin regarding the impacts of omega 3 and aspirin showed that the falls (27%) in the uterine artery PIs in the group receiving aspirin before and after intervention were 3.05 and 2.16; respectively (4). These differences were statistically significant. The falls (28%) in the uterine PIs in the group given aspirin plus omega 3 before and after intervention were 3.02 and 2.15; respectively. The finding was meaningful statistically. While in the group receiving only omega 3, the falls (16%) before and after intervention were 3.09 and 2.47; respectively, that was not statistically significant. The researchers found that aspirin per se or plus omega 3 decreased significantly the uterine artery PI, but they failed to show a significant association with omega 3 in this regard (4). The results obtained from this study show the effects of aspirin on reducing mean uterine PI was compatible with ours, but the fall in our study was lower. The difference could be elaborated as the selection of higher PI which caused significant differences in this regard, while in our study the inclusion criteria were PI>2.5. Additionally; in the study performed by Lazarrin, only the mean PI of two uterine arteries was investigated and each uterine artery PI was not studied individually. It has shown that low-dose aspirin treatment significantly improves ovarian responsiveness, uterine and ovarian blood flow velocity, and implantation and pregnancy rates in IVF patients. There were significant differences in the group receiving aspirin in comparison with the control group regarding ovarian follicles, the number of isolated oocytes, serum estradiol levels, uterine artery PI, and the rates of pregnancy (p<0.05). Uterine artery PI in the group receiving aspirin was 1.2, while it was 1.9 in the control group (17). The finding was compatible with ours. The current study was fulfilled on women with the history of two consecutive abortions when they were not pregnant, while other studies investigated the impacts of the drug during pregnancy. 

In another study has been performed on pregnant women with the history of three abortions regarding the effects of aspirin and heparin, it was determined that the women receiving heparin plus aspirin had lower rates of abortions but higher rates of live birth in comparison with those receiving aspirin per se. As shown in the present study, the protocol of combing two drugs provoked more therapeutic effects than single drug treatment (11). A study has been carried out to investigate the effects of vitamin E on endometrial thickness showed that consuming vitamin E for six months increased endometrial thickness and ultimately increased fertility rate (13). Unfortunately, the researchers did not investigate the uterine blood flow rates so we were unable to compare the results with ours. The current study showed that vitamin E had least beneficial efficacy so that it was able to produce only 2.5% fall in the mean PI of both uterine arteries. A study fulfilled by Takasaki and his collegues on the women having endometrial weakness and high radial artery resistance index demonstrated that administrating vitamin E decreased the resistance in 72% of the studied women, while L-arginine and sildenafil caused 89% and 92% decrease in the radial artery resistance (18).

## Conclusion

Vitamin E, aspirin and especially their combination are effective in improving uterine artery blood flow in women with recurrent abortion due to impaired uterine blood flow. it can be recommended as a new potential treatment for women with the history of recurrent abortions and impaired uterine blood flow. However, more well-designed studies are needed to find out whether the enhancement of uterine perfusion may lead to a better pregnancy outcome.

The current study demonstrated that co-administration of vitamin E and aspirin decreased uterine artery PI. Considering the results, it can be recommended to administer aspirin plus vitamin E for women with the history of recurrent abortions and impaired uterine blood flow.

## Note

***Registration ID in IRCT:*** ***IRCT2016022020035N2***
